# A New Benzothiazolthiazolidine Derivative, 11726172, Is Active *In Vitro*, *In Vivo*, and against Nonreplicating Cells of Mycobacterium tuberculosis

**DOI:** 10.1128/msphere.00369-22

**Published:** 2022-11-15

**Authors:** Elena G. Salina, Umberto Postiglione, Laurent R. Chiarelli, Deborah Recchia, Monika Záhorszká, Alexander Lepioshkin, Natalia Monakhova, Adrian Pál, Alessio Porta, Giuseppe Zanoni, Jana Korduláková, Elena Kazakova, Davide Sassera, Maria Rosalia Pasca, Vadim Makarov, Giulia Degiacomi

**Affiliations:** a Department of Biology and Biotechnology Lazzaro Spallanzani, University of Pavia, Pavia, Italy; b Bach Institute of Biochemistry, Research Centre of Biotechnology RAS, Moscow, Russia; c Department of Biochemistry, Faculty of Natural Sciences, Comenius University Bratislava, Bratislava, Slovakia; d Department of Chemistry, University of Pavia, Pavia, Italy; Antimicrobial Development Specialists, LLC

**Keywords:** *Mycobacterium tuberculosis*, antitubercular drug, copper, latency, nonreplicating cells

## Abstract

Tuberculosis (TB) still poses a global menace as one of the deadliest infectious diseases. A quarter of the human population is indeed latently infected with Mycobacterium tuberculosis. People with latent infection have a 5 to 10% lifetime risk of becoming ill with TB, representing a reservoir for TB active infection. This is a worrisome problem to overcome in the case of relapse; unfortunately, few drugs are effective against nonreplicating M. tuberculosis cells. Novel strategies to combat TB, including its latent form, are urgently needed. In response to the lack of new effective drugs and after screening about 500 original chemical molecules, we selected a compound, 11726172, that is endowed with potent antitubercular activity against M. tuberculosis both *in vitro* and *in vivo* and importantly also against dormant nonculturable bacilli. We also investigated the mechanism of action of 11726172 by applying a multidisciplinary approach, including transcriptomic, labeled metabolomic, biochemical, and microbiological procedures. Our results represent an important step forward in the development of a new antitubercular compound with a novel mechanism of action active against latent bacilli.

**IMPORTANCE** The discontinuation of TB services due to COVID-19 causes concern about a future resurgence of TB, also considering that latent infection affects a high number of people worldwide. To combat this situation, the identification of antitubercular compounds targeting Mycobacterium tuberculosis through novel mechanisms of action is necessary. These compounds should be active against not only replicating bacteria cells but also nonreplicating cells to limit the reservoir of latently infected people on which the bacterium can rely to spread after reactivation.

## INTRODUCTION

Mycobacterium tuberculosis, the etiological agent of tuberculosis (TB), ranked first as the most efficient single infectious agent for decades, claiming about 1.5 million lives every year (1). Just recently, COVID-19 dislodged TB from the first spot in the top 10 list of the deadliest infectious diseases worldwide. The WHO reported a decrease in global notifications of TB infections by 25 to 50% for the 3 months of the first COVID-19 wave and similar decreases corresponding to subsequent waves because of the disruption of TB services (1–3). This situation raises concerns of future rebounds, increasing the risk of multidrug-resistant TB (MDR-TB). Drug resistance is indeed a growing threat to manage, considering that one in three deaths from antimicrobial resistance is due to drug-resistant forms of TB (4). Current treatment regimens for TB require combinations of multiple drugs, with durations ranging from 6 months for drug-susceptible TB to a typical duration of 6 to 20 months for MDR-TB but possibly longer in the case of additional drug resistance (5). Globally, the latest available data show treatment success rates of 85% for drug-susceptible TB and 57% for MDR or rifampin-resistant TB (1). Thus, TB remains a global health menace worldwide.

Furthermore, a quarter of the human population is latently infected with M. tuberculosis without overt symptoms of disease and the ability to transmit it (1). People with latent infection have a 5 to 10% lifetime risk of falling ill with TB, and those with compromised immune systems have an even a higher risk of disease progression. So, latent TB represents a reservoir for TB active infection (6, 7). Unfortunately, few drugs are effective against M. tuberculosis nonreplicating cells, which also represents a worrisome issue to overcome in the case of relapse. Current therapy for latent TB includes only three drugs, including isoniazid, rifampin, and rifapentine (7). The three possible regimens are the following: 3 months of once-weekly isoniazid plus rifapentine, 4 months of daily rifampin, and 3 months of daily isoniazid plus rifampin (8). An alternative monotherapy is 6 or 9 months of daily isoniazid (8). It is important to underline that isoniazid is not active against M. tuberculosis nonreplicating cells because it targets cell wall biosynthesis; most probably, this drug is able to kill sensitive, actively growing bacilli. Rifampin and rifapentine target transcription by binding to the β-subunit of RNA polymerase, once again a pathway more important for metabolically active cells (6).

The last three antitubercular drugs to be introduced in clinical settings are active against latent TB: bedaquiline, delamanid, and pretomanid. Bedaquiline targets mycobacterial F_o_F_1_ ATP synthase, which is essential for mycobacterial survival during the TB latent state; for this reason, bedaquiline is highly active against nonreplicating mycobacteria (9). Recently, a new generation of bedaquiline derivatives, such as TBAJ-587 and WX-081, entered phase I and II clinical trials (10, 11), creating the possibility of new compounds active against dormant TB. Pretomanid and delamanid belong to the nitroimidazole class and are prodrugs activated by deazaflavin-dependent nitroreductase (Ddn). Upon their activation, the des-nitro metabolite and reactive nitrogen species, primarily nitric oxide (NO), are generated and are responsible for the activity of these compounds against nonreplicating M. tuberculosis bacilli (12).

In this context, there is a pressing need to search for new, highly effective, low-toxicity drugs with novel targets that have the potential both to shorten the duration of treatment, particularly for drug-resistant forms of TB, and to be active against latent infections.

In this work, following a screening of about 500 original chemical molecules, we selected 4-nitro-2,1,3-benzothiadiazol-5-yl-1,3-thiazolidine-3-carbodithioate compound (11726172), which displays a potent antitubercular activity against M. tuberculosis both *in vitro* and *in vivo* and also against dormant nonculturable (NC) bacilli.

Additionally, the mechanism of action of 11726172 was investigated using a multidisciplinary approach, including transcriptomic, labeled metabolomic, biochemical, and microbiological procedures.

## RESULTS

### Activity of 11726172.

We screened about 500 original chemical compounds synthesized at the Research Center of Biotechnology RAS in a search of hits endowed with antitubercular activity (data not shown). These compounds belong to various chemical classes, mainly polysubstituted heterocycles. All compounds were tested by a resazurin microtiter assay (REMA) (13) at a final concentration of 40 μg/mL.

In this screen, 4-nitro-2,1,3-benzothiadiazol-5-yl-1,3-thiazolidine-3-carbodithioate (11726172) exhibited an MIC of 0.25 μg/mL and therefore was selected for further study.

### 11726172 is bactericidal against NC M. tuberculosis.

The activity of 11726172 was further studied against dormant NC *M.tuberculosis* H37Rv bacilli with a “zero-CFU” phenotype, obtained under potassium deficiency (14, 15). This approach using an *in vitro* model that mimics latency has been previously validated and reported (16–19).

The NC bacilli were characterized by a high recovery potential of being able to resuscitate into a fully culturable state after reintroduction of potassium ions (14, 15). As these NC cells are unable to produce colonies on nonselective agar-solidified medium, we applied the most probable number (MPN) assay to liquid medium to check the effect of 11726172 on the viability of dormant NC cells.

**FIG 1:**
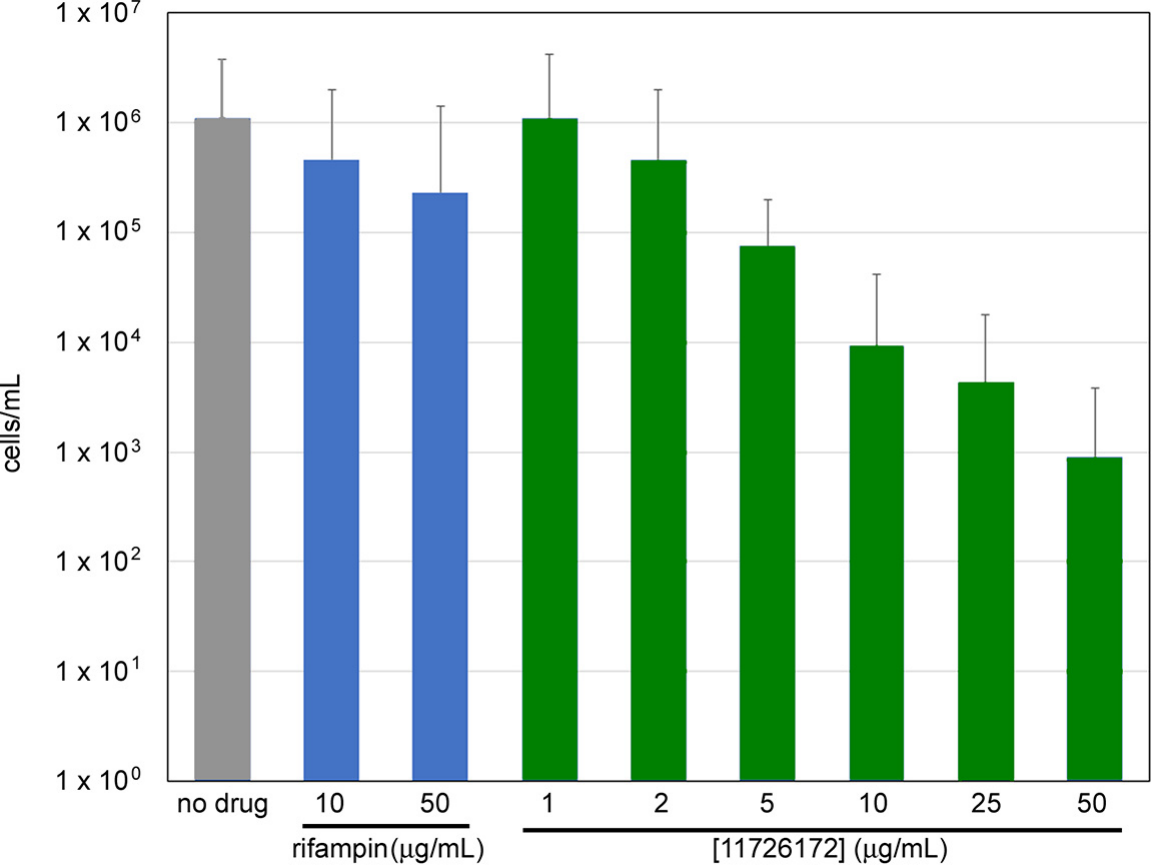
Dose-dependent escalation of bactericidal activity of 11726172 for dormant nonreplicating M. tuberculosis H37Rv. The experiment was repeated three times with comparable results. The figure shows one representative experiment.

Incubation of dormant NC cells with 11726172 at different concentrations resulted in dose-dependent escalation of killing activity. In fact, 11726172 started to kill NC bacilli at a concentration of 2 μg/mL (causing a 0.5-log decrease in cell viability), with the maximal effect observed at 50 μg/mL with a 3-log decrease in cell viability (Fig. 1), while rifampin at a concentration up to 50 μg/mL has almost no effect on the viability of dormant NC bacteria (less than 1 log) (Fig. 1). Thus, our results show that 11726172 is able to inhibit the growth of both replicating and nonreplicating M. tuberculosis H37Rv cells.

### SAR study of 11726172.

To understand the substituent requirements needed to achieve activity against M. tuberculosis, structure-activity relationship (SAR) studies were performed (Table 1).

**TABLE 1:** Activity of 11726172 and its derivatives against M. tuberculosis H37Rv growth

Compounds	Molecular structure	MIC (μg/ml)
11726172	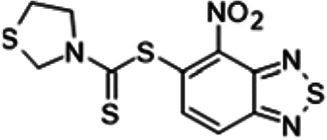	0.25
11726170	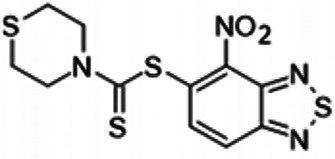	2,5
11726171	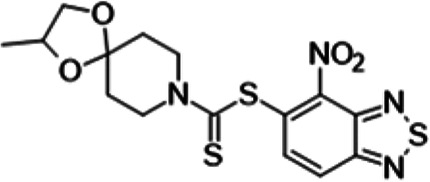	10
11726173	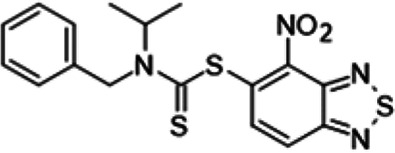	10
11726174	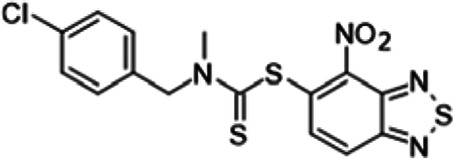	>20
11726175	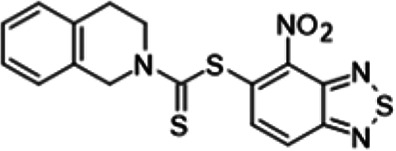	10
11726176	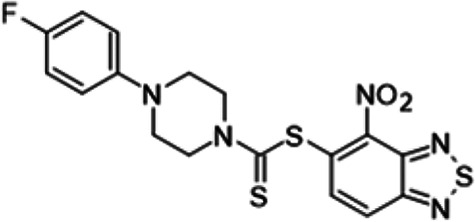	>20
11726177	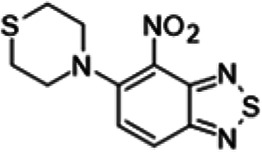	20
11726178	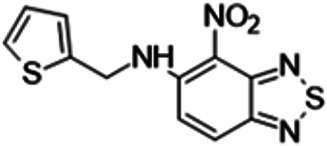	>20
11726179	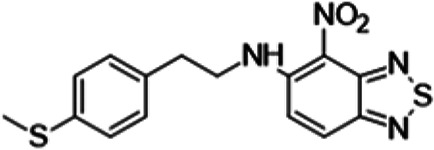	>20
11726180	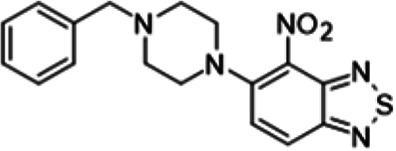	>20
11726181	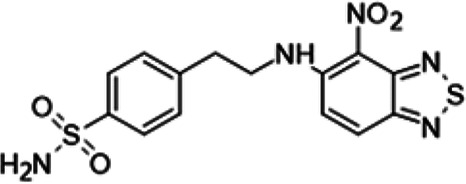	>20
Streptomycin		0.25

Eleven derivatives of compound 11726172 were designed and synthesized, but no other active compounds were identified. However, we observed that the dithiocarbamoyl moiety is one of the essential fragments in the molecule, because derivatives lacking it were completely inactive (11726177 to 11726181). Compounds with unsaturated substitutes (11726174 to 11726176) were also much less active or completely inactive. The lead compound, 11726172, is the smallest compound in the series, and the sulfur-containing ring has a positive influence on antimycobacterial activity. Therefore, we can conclude that sulfur and the dithiocarbamoyl moiety play important roles in the mode of action of the studied compounds.

### Study of the mechanism of action of 11726172.

To study the mechanism of action of the novel compound, we first performed a classical approach. Mutations in the gene encoding the putative cellular target of the drug confer resistance, thus helping in the investigation of the mechanism of action of antimicrobials (20, 21). In this case, despite many attempts, we were not able to select any M. tuberculosis H37Rv drug-resistant mutants to identify the 11726172 molecular target (data not shown).

As a second strategy for discovering the mechanism of action, we used a panel of M. tuberculosis mutant strains resistant to known drugs, carrying known mutations in genes encoding targets (*dprE1*, *mmpL3*, *atpE*, *pyrG*, *panK*, etc.), activators (*ethA*, *mrx2*), and repressors of efflux pumps (*Rv0678*, etc.) (Table 2) (16, 20, 22–27). The activity of 11726172 was evaluated against these mutants, but the strains were all found to be as sensitive to the compound as the M. tuberculosis H37Rv wild-type strain. Interestingly, this compound is also highly effective against two MDR M. tuberculosis clinical isolates (Table 2).

**TABLE 2:** Activity of 11726172 against a panel of M. tuberculosis strains

Strain^*a*^	MIC (μg/mL)		Reference
	11726172	INH	
H37Rv	0.25	0.05	
IC1 (resistant to STR, INH, RIF, EMB)	0.25	>2	22
IC2 (resistant to STR, INH, RIF, EMB, PYR, ETH, CM)	0.25	>2	22
53.3 (Rv2466c, W28S)	0.25	0.05	16
53.8 (Rv0579, L240V; Rv0158, V48A; esxD, M41R)	0.25	0.05	23
NTB1 (DprE1, G387S)	0.25	0.05	24
DR1 (Mmpl3, V681I)	0.25	0.05	25
Ty1 (Rv3405c, c190t)	0.25	0.05	26
88.1 (CoaA, Q207R)	0.25	0.05	27
88.7 (PyrG, V186G)	0.25	0.05	20
81.10 (EthA, D1109-37)	0.25	0.05	20

a STR, streptomycin; INH, isoniazid; RIF, rifampin; EMB, ethambutol; PYR, pyrazinamide; ETH, ethionamide; CM, capreomycin.

**FIG 2:**
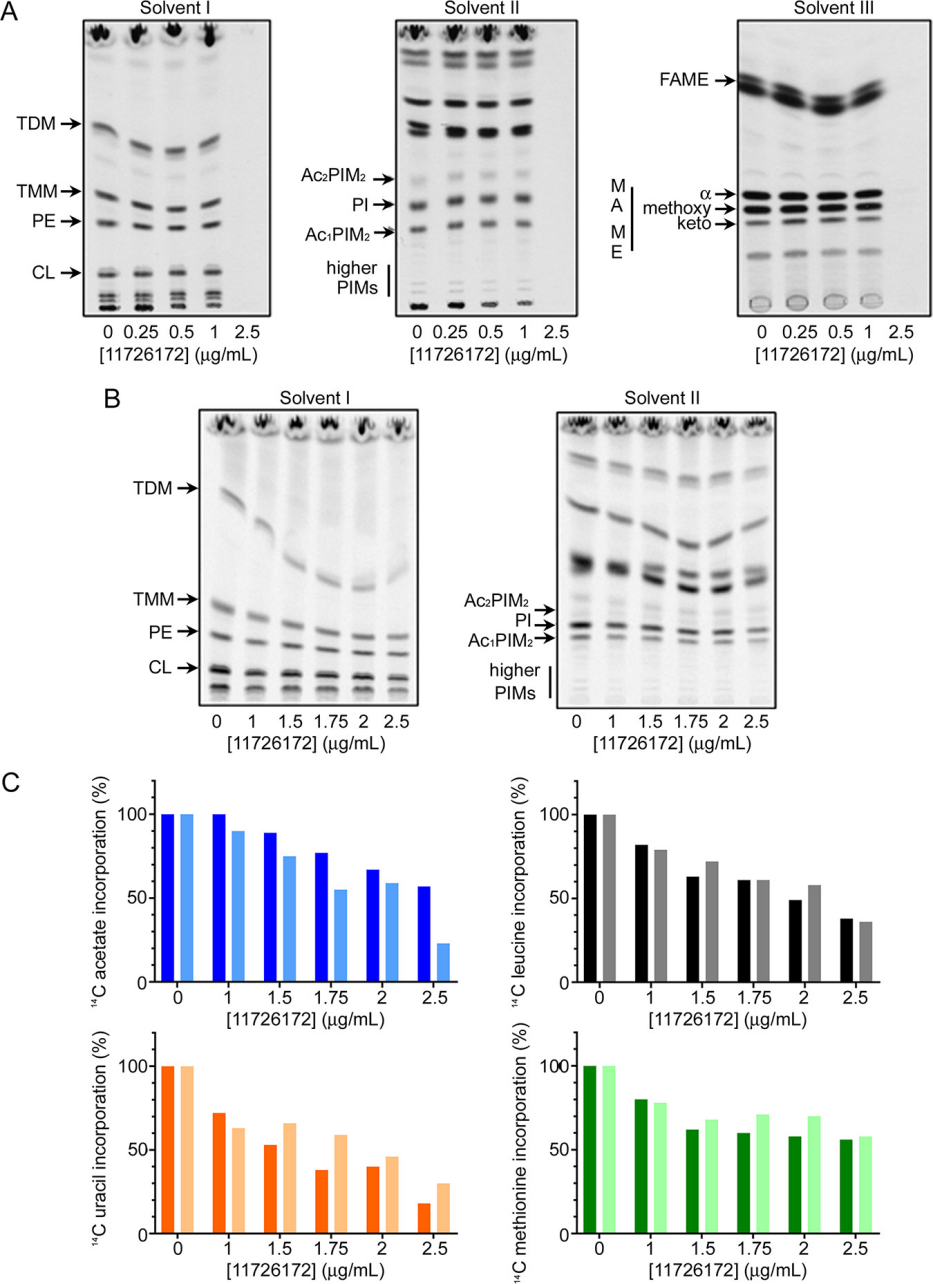
Metabolic labeling of M. tuberculosis H37Rv and M. tuberculosis H37Ra cells treated with compound 11726172. (A) Lipid analysis of M. tuberculosis H37Rv cells grown in the presence of 11726172. [^14^C]acetate (in a final concentration of 0.5 μCi/mL) was added to the culture in the presence of increasing concentrations of 11726172. The lipids were extracted and analyzed by TLC and detected by autoradiography. (B) Lipid analysis of M. tuberculosis H37Ra cells grown in the presence of 11726172. The cells were grown and metabolically labeled, and the lipids were analyzed as described above. The lipids were visualized using an Amersham Typhoon biomolecular imager. (C) Macromolecular synthesis assay with M. tuberculosis H37Ra treated with 11726172. [^14^C]acetate (blue), [^14^C]leucine (gray), [^14^C]uracil (orange), and [^14^C]methionine (green) were added as described above at a final concentration of 0.5 μCi/mL. After 24 h of incorporation, ^14^C was quantified by scintillation spectrometry. Data from two independent experiments are shown by two different color shades. TDM, trehalose dimycolates; TMM, trehalose monomycolates; PE, phosphatidylethanolamine; CL, cardiolipin; PIM, phosphatidylinositol mannosides; AcPIM, acylated forms of phosphatidylinositol mannosides; FAME, fatty acid methyl esters; MAME, mycolic acid methyl esters.

Furthermore, as a third approach, we evaluated whether the synthesis of macromolecules as well as lipids was affected after 11726172 exposure. Treatment of M. tuberculosis H37Rv cells with up to 2.5 μg/mL of 11726172 did not result in significant specific changes in the lipid and mycolic acid profiles; only an overall decrease of ^14^C incorporation was observed (Fig. 2A). Similarly, treatment of M. tuberculosis H37Ra cells with up to 2.5 μg/mL of 11726172 did not lead to any specific changes in lipid or macromolecule composition (Fig. 2B and C).

Unfortunately, all of the attempts pursued for elucidating the mechanism of action of 11726172 were unsuccessful, suggesting that the cellular target is so essential for mycobacterial cell growth that it cannot be mutated. Therefore, alternative strategies were used to gain more insights about the 11726172 mechanism of action.

### Transcriptome analysis of M. tuberculosis H37Rv exposed to 11726172.

An alternative method used to study the mechanism of action of drugs is the characterization of the global gene expression pattern of M. tuberculosis H37Rv in response to drug treatment (28, 29). Therefore, transcriptome analysis of M. tuberculosis H37Rv exposed to 11726172 at concentrations of 10× MIC (2.5 μg/mL) and 30× MIC (7.5 μg/mL) was performed by RNA sequencing (RNA-seq). As a control, untreated M. tuberculosis H37Rv cultures were used. Three biological replicates for each condition were performed. Sequencing and mapping were successful, resulting in 23.7 ± 9.2 million reads mapped per sample.

**FIG 3:**
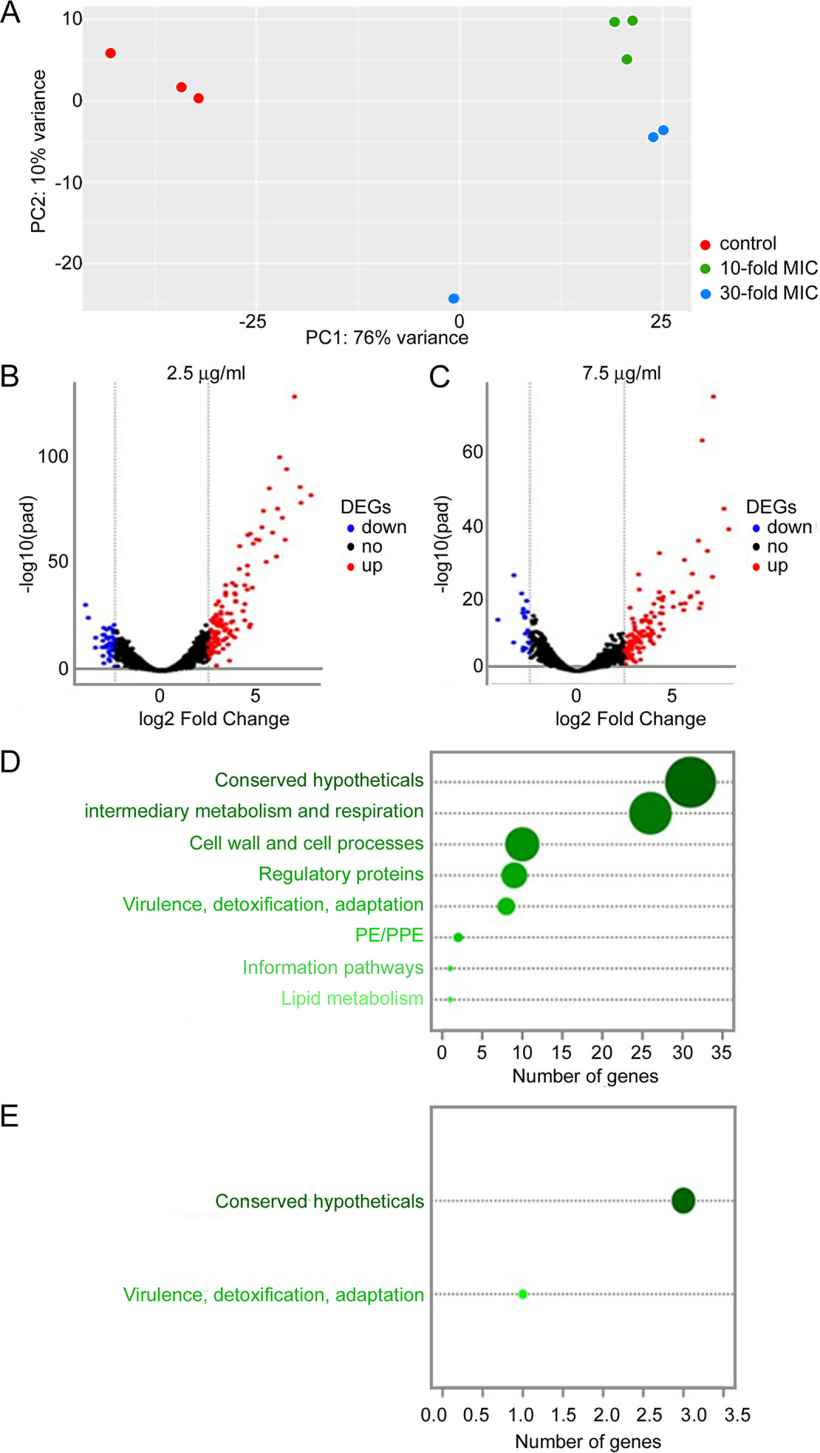
Transcriptome analysis of M. tuberculosis H37Rv cells exposed to 11726172. (A) Principal-component analysis (PCA) plot of M. tuberculosis H37Rv samples (control [not treated], 10× MIC, and 30× MIC). (B) Volcano plots of differentially expressed genes (10× MIC, 2.5 μg/mL). (C) Volcano plots of differentially expressed genes (30× MIC, 7.5 μg/mL). (D) Functional characterization of genes overexpressed in both treatment conditions. (E) Functional characterization of genes underexpressed in both treatment conditions.

As an initial control of the data set, the overall similarity between and within groups of samples was assessed using a principal-component analysis (PCA) (Fig. 3A). The untreated (control group) samples are well separated from the treated samples (10× MIC and 30× MIC groups) along the first direction (PC1), which represents 76% of the total variance. Instead, the 10× MIC and 30× MIC samples appear to be best separated along the second direction (PC2), which collects only 10% of the variance (Fig. 3A). These results confirm the solidity of the project’s experimental design.

Out of 3,906 coding DNA sequences, 167 (4.2%) and 146 (3.7%) genes were found to be differentially expressed in M. tuberculosis H37Rv cells treated with 10× MIC and 30× MIC of 11726172, respectively, compared to the control group. In the case of the 10× MIC group, 126 genes were upregulated with respect to the control (2.5- to 7.99-fold), while 41 genes were underexpressed (2.5- to 4.07-fold) (Fig. 3B). On the other hand, in the case of the 30× MIC group, the number of upregulated genes was 127 (2.5- to 8.02-fold), while the number of downregulated genes was 19 (2.5- to 4.19-fold) (Fig. 3C). Overall, 88 genes were found to be commonly upregulated in both conditions, while 4 genes were repressed (see Table S1 in the supplemental material). All these results were confirmed by meta-analyses of RNA-seq using the MetaRNA-Seq package in R.

10.1128/msphere.00369-22.1 TABLE S1 In Table S1, 88 differentially expressed genes in both *M. tuberculosis* H37Rv samples treated with 10× MIC or 30 MIC of 11726172 are shown. Download Table S1, DOCX file, 0.03 MB. Copyright © 2022 Salina et al. 2022 Salina et al. https://creativecommons.org/licenses/by/4.0/ This content is distributed under the terms of the Creative Commons Attribution 4.0 International license.

For these genes, a functional annotation analysis was performed using both the DAVID functional annotation analysis tool and TubercuList, and the following pathways (false discovery rate [FDR] < 0.05) were found to be most enriched: (i) response to copper ion (GO:0046688), (ii) cysteine biosynthetic process (GO0019344), (iii) response to cadmium ion (GO:0046686), (iv) protein disulfide oxidoreductase activity (GO:0015035), and (v) cysteine biosynthetic process from serine (GO:0006535).

In parallel, assignment of M. tuberculosis H37Rv genes to functional categories was also performed based on the TubercuList (30, 31). Genes that were overexpressed in both treatments were found to belong to eight categories. The categories with the most numerous genes were “conserved hypotheticals” and “intermediary metabolism and respiration.” Interestingly, several genes known to be induced by metals were upregulated in response to 11726172 exposure, such as *cadI* (encoding a putative metal transporter), *furA* (encoding a ferric uptake regulation protein), and *cysK2* (encoding a protein involved in mycothiol biosynthesis) (Fig. 3D).

One of the more strongly induced genes present in “intermediary metabolism and respiration” category is *cyp135A1*, which codes for a cytochrome P450. This specific Cyp135A1 could be involved in the detoxification process correlated with 11726172. Another induced gene in this category is *trxC*, coding for a thioredoxin.

Overexpression of both *cyp135A1* and *trxC* in M. tuberculosis exposed to 11726172 under both conditions studied was confirmed by quantitative real-time PCR (qRT-PCR) (Table 3).

**TABLE 3:** Evaluation of expression levels of *cyp135A1*, *trxC*, and *mazE8* genes by qPCR

Treatment of M. tuberculosis cultures	Expression level		
	*cyp135A1*	*trxC*	*Rv2274A*
10× MIC	8.86 ± 0.39	10.65 ± 0.60	0.22 ± 1.48
30× MIC	6.57 ± 0.12	5.17 ± 1.24	–4.55 ± 1.97

In the “information pathway” category, the most strongly induced gene was *rshA* (Table S1), coding for an anti-sigma factor which inhibits the sigma factor *sigH*.

Four genes were underexpressed in both treatments: three belonged to the “conserved hypothetical” functional category, while one was part of the “virulence, detoxification, and adaptation” category. The most repressed gene was *Rv2274A*, coding for the antitoxin MazE8 (Fig. 3E).

Underexpression of *Rv2274A* in M. tuberculosis exposed to 11726172 under both conditions was confirmed by qRT-PCR (Table 3).

Taken together, these data suggest that 11726172 could have a pleiotropic effect on M. tuberculosis bacteria, triggering general stress responses. In particular, it seems to affect cell permeability, consequently perturbing metal homeostasis as well as cytoplasmic redox potential.

### The activity of 11726172 is copper related.

Because the transcriptomic data suggested that 11726172 treatment could affect metal homeostasis in M. tuberculosis bacilli, the correlation of activity with the presence of metal ions was further investigated.

**FIG 4:**
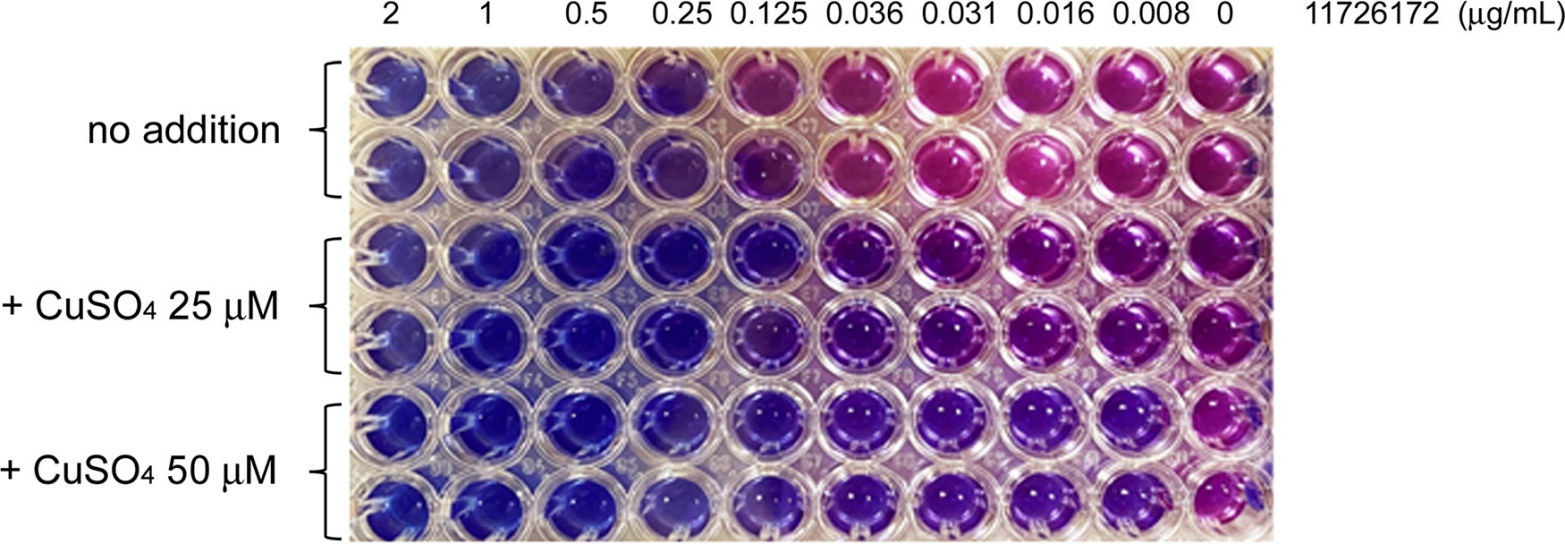
Copper ions gradually increase the activity of 1726172 against M. tuberculosis.

To this aim, the activity of 11726172 against M. tuberculosis H37Rv growth was determined using Sauton/oleic acid-albumin-dextrose-catalase (OADC) medium in the presence of increasing concentrations of metal cations such as Cu^2+^, Ni^2+^, Co^2+^, and Zn^2+^. 11726172 was found to be significantly more active against M. tuberculosis H37Rv growth in the presence of copper ions, with MIC values lowered 2- to 4-fold in the presence of 25 to 50 μM Cu^2+^ (Table 4; Fig. 4). In contrast, Ni^2+^, Co^2+^, and Zn^2+^ ions were not able to affect the 11726172 antitubercular activity at concentrations up to 50 μM.

**TABLE 4:** Activity of 11726172 in the presence of metal cations against M. tuberculosis H37Rv

Metal cation concentration (μM)	MIC for M. tuberculosis H37Rv (μg/mL) in the presence of:			
	Cu^2+^	Ni^2+^	Co^2+^	Zn^2+^
0	0.5	0.5	0.5	0.5
10	0.25	0.5	0.5	0.5
25	0.25	0.5	0.5	0.5
50	0.125	0.5	0.5	0.5

**FIG 5:**
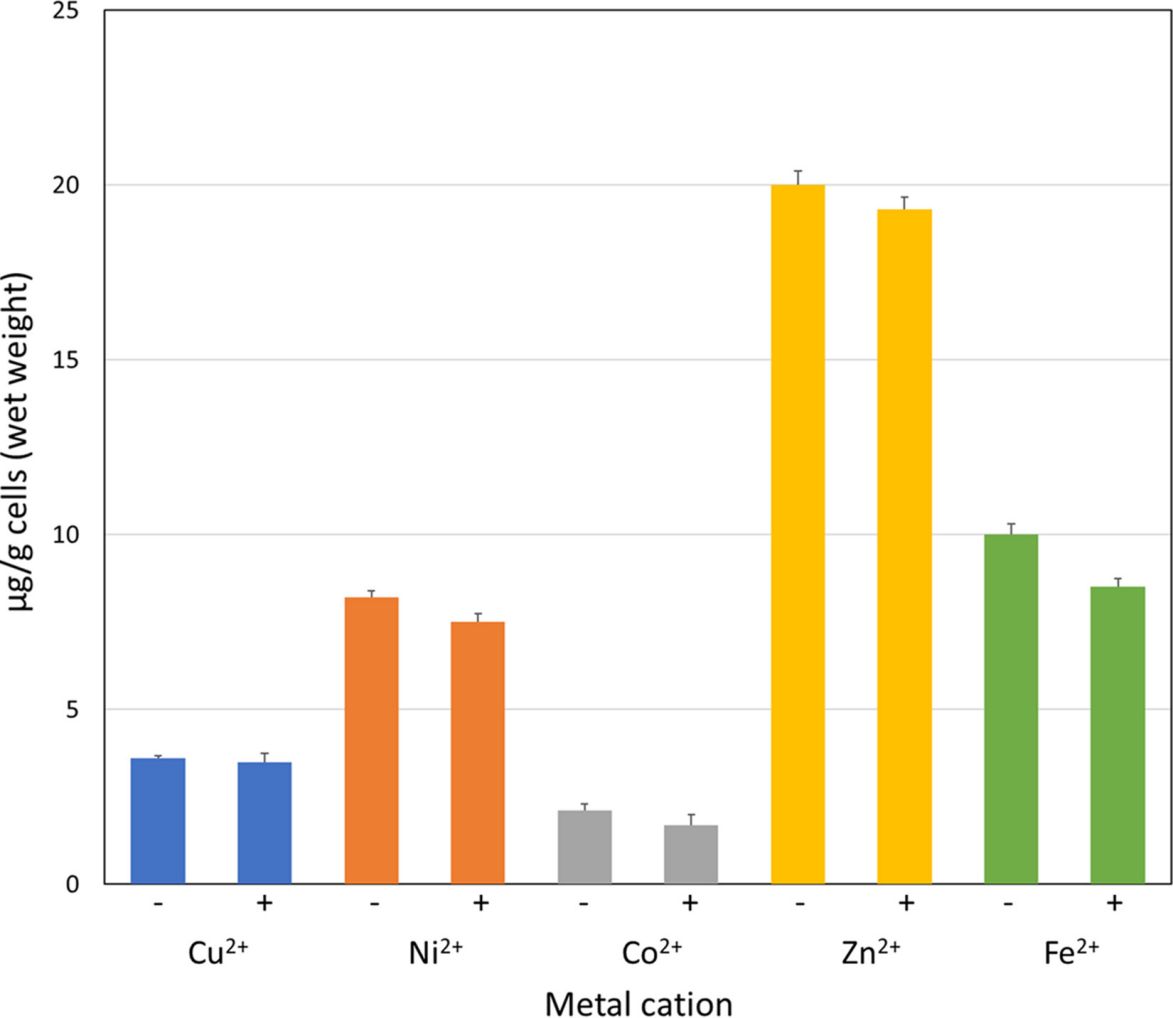
Analysis of metal content in M. tuberculosis H37Rv cell lysates after treatment with 11726172. Determination of intracellular content of Cu^2+^, Ni^2+^, Co^2+^, Zn^2+^, and Fe^2+^ after incubation with a 50 μM concentration of the respective ion in the absence (−) or presence (+) of 7.5 μg/mL 11726172.

To investigate whether the mechanism of action of 11726172 is connected to the accumulation of toxic concentrations of metals, and particularly of copper, inside mycobacterial cells, bacilli were grown in Sauton medium supplemented with Tween 80 and OADC in the presence of 50 μM CuSO_4_, NiSO_4_, CoCl_2_, and ZnSO_4_, with or without 11726172, using dimethyl sulfoxide (DMSO) (0.1%) as a reference. As shown in Fig. 5, the presence of 11726172 did not influence the accumulation of metal cations in mycobacterial cells.

In conclusion, we demonstrated that the antitubercular activity of 11726172 is enhanced by the presence of copper even if the compound does not determine a toxic metal accumulation in M. tuberculosis H37Rv cells. So, it could be hypothesized that either the cellular target or a possible activator of this compound is copper dependent.

### Metabolic transformation of 11726172 upon contact with Mycobacterium bovis BCG cells.

**FIG 6:**
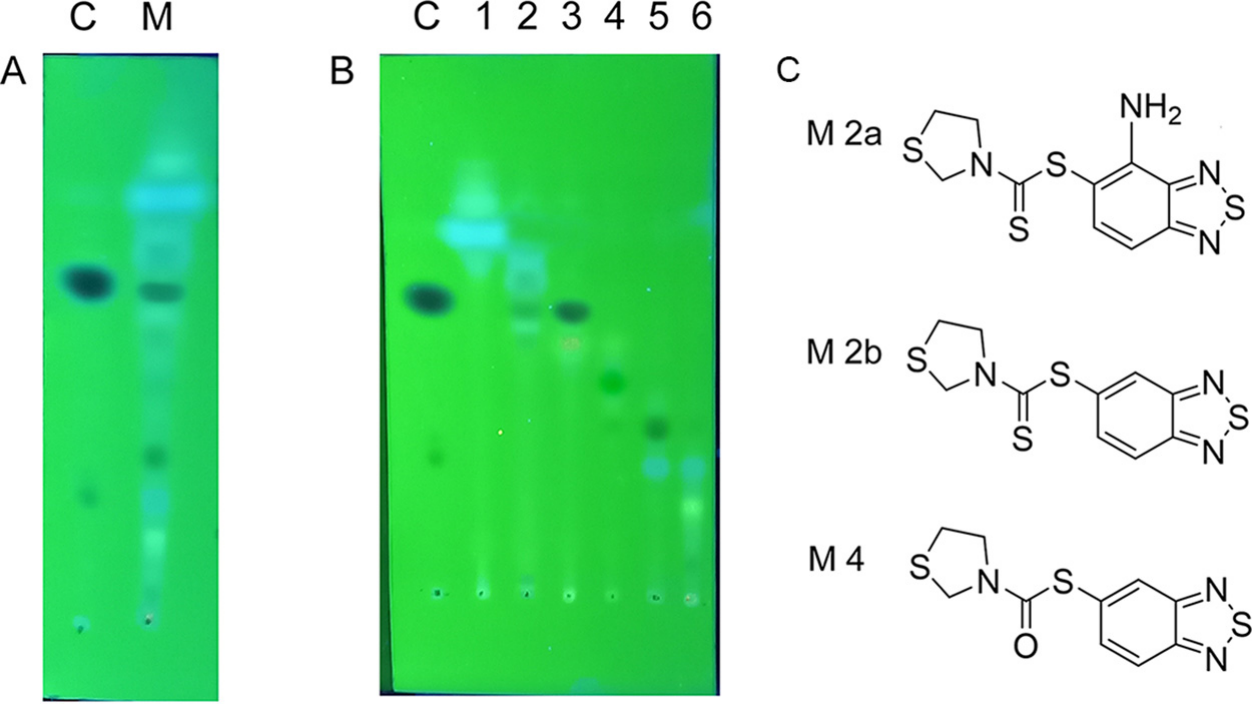
Identification of the potential 11726172 metabolites produced in M. bovis cells. (A) TLC analysis (hexane-ethyl acetate at a 6:4 ratio, visualized under UV light) of the compound (C) and of the metabolite mixture extracted in chloroform after incubation with mycobacterial culture (M). (B) TLC analysis of chromatographic fractions 1 to 6 from the cell culture extract. (C) Structure of potential 11726172 metabolites M 2a, M 2b, and M 4 identified by HESI-MS analysis (as shown in Fig. 7).

To understand how M. tuberculosis could metabolize 11726172, and the effects of such transformation on its activity, 10 mg of this compound (corresponding to 100× MIC) was added to a 200-mL M. bovis BCG culture in exponential phase and incubated at 37°C for 16 h. The culture was then extracted with chloroform, and the organic phase was analyzed by thin-layer chromatography (TLC). After 16 h of incubation, 11726172 was partially metabolized into different compounds, but the main spot in the chromatogram remained that of 11726172 (Fig. 6, fraction 3). Then, to characterize the products metabolized into mycobacterial cells, they were separated by silica gel flash column chromatography, and the isolated fractions were subjected to heated electrospray ionization mass spectrometry HESI-MS analysis for identification. Furthermore, for each fraction, the antimycobacterial activity was assessed.

**FIG 7:**
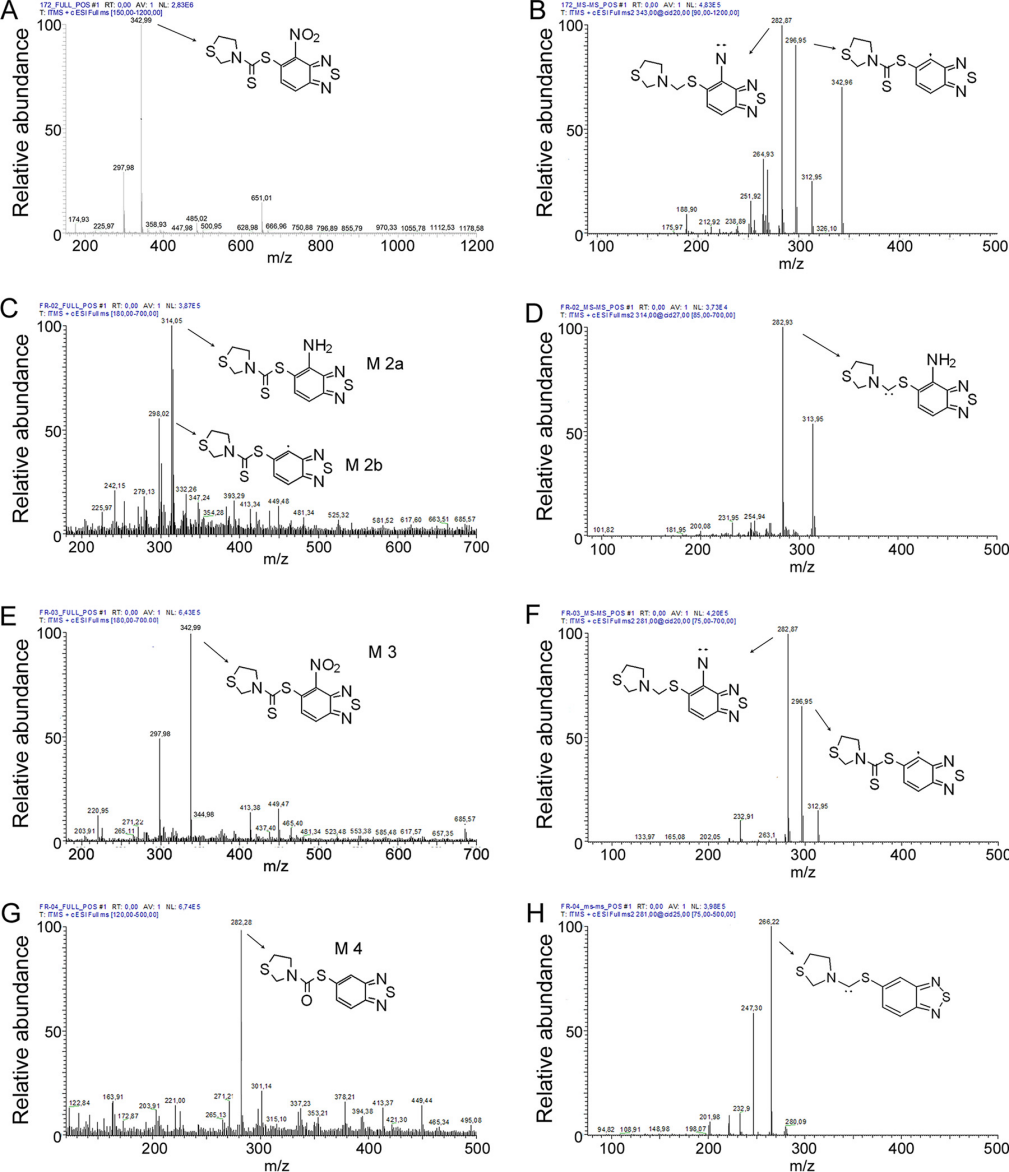
HESI-MS (positive-mode) direct infusion analysis of the silica gel chromatography fractions of 11726172-treated M. bovis culture extract. (A) Full mass of fraction 1; (B) fragmentation pattern of fraction 1; (C) full mass of fraction 2; (D) fragmentation pattern of M 2a; (E) full mass of fraction 3; (F) fragmentation pattern of M 3; (G) full mass of fraction 4; (H) fragmentation pattern of M 4.

For three out of six isolated fractions, mass spectral analysis allowed the determination of the structure of the metabolites. In particular, we confirmed that the main metabolite that eluted in fraction 3 (M 3) corresponded to nonmetabolized 11726172 (Fig. 6 and 7). Regarding the other identified metabolites (M 2a and M 2b eluted in fraction 2 and M 4 eluted in fraction 4), mass spectra revealed that the main transformations occurred at the level of the nitro group through reduction to amine or removal or at the level of the sulfur atoms of the carbodithioate moiety (Fig. 7). However, all the transformations resulted in inactivation of the compound, as only fraction 3 displayed antimycobacterial activity, further confirming the essentiality of the dithiocarbamoyl moiety and highlighting the importance of the nitro group. Moreover, it was demonstrated that 11726172 is probably the true active compound and not a prodrug. This result thus indicates that 11726172 is relatively stable inside mycobacterial cells and directly active against M. tuberculosis.

### Drug combinations with 11726172 tested by CBA.

To study the possible inclusion of 11726172 in combinatorial antitubercular therapy, a checkerboard assay (CBA) was performed.

First, the activity of each individual compound chosen among first-line and recently approved drugs (ethionamide, isoniazid [INH], moxifloxacin, and bedaquiline) was determined by REMA (MIC values as follows: bedaquiline, 0,03 μg/mL; ethambutol, 1 μg/mL; linezolid, 0,5 μg/mL; moxifloxacin, 0,06 μg/mL; isoniazid, 0,06 μg/mL). Then, to evaluate and quantify pairwise drug interactions, CBAs were performed, using growth inhibition as the endpoint readout. The calculated fractional inhibitory concentration index (FICI), which is the main indicator of synergy, was >0.5 to 4 for each assayed antibiotic, indicating no antagonism interaction with 11726172 and, thus, the suitability of this compound for possible combinational therapy.

### Studies in a mouse model of acute TB.

We tested the efficacy of 11726172 treatment in a murine model of M. tuberculosis acute infection. To this aim, male BALB/c/Cit mice were infected by intravenous injection of M. tuberculosis Н37Rv at a concentration of 5 × 10^6^ CFU/mouse into the lateral tail vein. One week after M. tuberculosis H37Rv infection, mice were treated 5 times per week for 4 consecutive weeks by gastric administration of either 11726172 (at 100 mg/kg) or INH (at 25 mg/kg). After 30 days, the mice were sacrificed, and the total lung was processed for microbiological analysis. Results showed a significant reduction in pulmonary mycobacterial burden, with about a 2-log reduction of the mean pulmonary CFU reached after 28 days of treatment (*P* value of 0.012) in the group receiving 11726172 (Table 5).

**TABLE 5:** *In vivo* efficacy studies^*a*^

Treatment for animal group	Survival (day postinfection)	Log CFU	Log CFU reduction
INH (25 mg/kg)	No deaths	3.90 ± 0.32	5.76
11726172 (100 mg/kg)	No deaths	7.25 ± 0.17	2.41
No treatment	23.17 ± 0.75	9.66 ± 0.11	

a The efficacy of 11726172 in a mouse model of acute M. tuberculosis infection was compared with that of INH.

After 11726172 treatment, all mice were alive but looked ill and stooped or bent over. We observed 5 to 7 visible foci of infection in each lung of the mice, and the spleen was enlarged 2.5-fold. On the other hand, all mice from the negative-control group (No treatment in Table 5) died after 23 days postinfection with TB typical lesions as large foci in the lungs and 3- to 4-fold enlarged spleens.

In conclusion, the compound is discretely active in a murine model of acute TB with a log CFU reduction of 2.41.

## DISCUSSION

After a long period without new antitubercular compounds on the horizon, TB drug discovery presents many novel drug candidates in clinical trials, although only a few of them successfully progress to clinical development (32).

A new antitubercular drug should be (i) active *in vitro*, (ii) active *in vivo*, (iii) active against M. tuberculosis nonreplicating cells, (iv) active against M. tuberculosis drug-resistant strains (including MDR), and (v) suitable for use in drug combinations.

The combinational drug treatment for multidrug-resistant/rifampin-resistant tuberculosis (MDR/RR-TB) is characterized by adverse events, toxicity, long duration, and poor effectiveness. Therefore, it is mandatory to find new antitubercular compounds that can be used in combination with existing drugs. For this reason, several clinical trials on combination therapy are under way. Recently, it was shown in a randomized trial that 4 months of antitubercular treatment with first-line drugs was as effective as 6 months of treatment in 1,204 children with drug-susceptible nonsevere tuberculosis (33). In the NIX trial, the combination of bedaquiline, pretomanid, and linezolid was successful in most patients with highly drug-resistant tuberculosis (34).

To avoid a possible relapse of TB, an antitubercular drug should be active against nonreplicating cells of M. tuberculosis. In addition, such a drug could also be used to prevent the onset of active TB in patients with the latent form of TB. Interestingly, the three newly approved antitubercular drugs (bedaquiline, delamanid, and pretomanid) are active against nonreplicating cells (35), but several M. tuberculosis clinical isolates resistant to these antibiotics are already circulating. Consequently, new drugs with new mechanisms of action are needed.

In this work, we found that 11726172 is active against M. tuberculosis growth both *in vitro* and *in vivo*; furthermore, it is also active against nonreplicating cells and could be used in combination with antitubercular drugs.

We also showed that the compound is active against MDR clinical isolates as well as M. tuberculosis mutants harboring mutations in genes encoding known targets, activators, etc. Notably, this finding underlines that 11726172 has a new mechanism of action. Another crucial point is that we were unable to isolate mutants resistant to 11726172. Since the drug is active on replicating and nonreplicating bacteria, the reason for the lack of isolation of mutants might be that it inhibits multiple targets or that the target is very essential and probably indispensable for nonreplicating bacteria and therefore not selectable under growth-permitting conditions. This can be a great advantage for eventual introduction into the clinical setting.

The use of transcriptomic analysis for understanding a drug’s mode of action provides useful insights into the target pathways affected by the action of the compound (36). Thanks to the contribution of transcriptomics, we showed that 11726172 treatment perturbs both the metal homeostasis and the cytoplasmic redox potential in mycobacterial cells.

Among the induced genes, we found those coding for several proteins that are induced in the presence of cellular stress, such as the following. Cytochromes P450 are a group of heme-thiolate monooxygenases which oxidize a variety of structurally unrelated compounds, including steroids, fatty acids, and xenobiotics. RshA is probably involved in survival following heat shock and oxidative stress and could be induced by the stress caused by drug exposure. *Rv2274A*, coding for the antitoxin MazE8, was downregulated, while all the toxins of M. tuberculosis were induced. M. tuberculosis has multiple toxin-antitoxin systems that are involved in regulating adaptive responses to stresses associated with the host environment and drug treatment (37). In our case, repression of the antitoxin MazE8 might help the compound exert its toxic effect against M. tuberculosis cells.

Metal-induced genes can be very important; in fact, they may act as regulators of drug tolerance (38). Our compound affects metal homeostasis, revealing a possible relation with metal ions.

In particular, M. tuberculosis has a protection mechanism in response to higher concentrations of copper that increases the formation of toxic hydroxyl radicals from hydrogen peroxide, a reaction that is catalyzed by Cu(I) (39). It was previously demonstrated that strong induction of the *csoR* and *ctpV* genes, which were found to be overexpressed in the transcriptome analysis upon treatment with 11726172, was correlated with increased levels of exclusively Cu^2+^ cations, demonstrating the specificity of the induction of these genes by copper (40). CtpV is an inner membrane transporter that probably functions as an efflux pump moving copper out of the cell. Strong induction of the *csoR* and *ctpV* genes in response to increasing levels of Cu^2+^ was demonstrated recently for 1-hydroxy-5-*R*-pyridine-2(1*H*)-thiones, Cu-dependent inhibitors of M. tuberculosis that accumulate toxic concentrations of copper inside treated bacilli (41). In response to 11726172 treatment, *mymT*, the gene encoding cytoplasmic Cu^2+^-binding metallothionein, which plays another important role in Cu^2+^ homeostasis in M. tuberculosis, was also induced.

Interestingly, only the presence of copper increases the activity of 11726172. Recently, copper has been identified as an important player in innate immunity (42). In particular, copper levels in the macrophage phagosome as well as serum and tissues may rapidly increase in response to M. tuberculosis infection up to 400 μM (39, 43, 44). We could not find an influence of 11726172 on the accumulation of a toxic concentration of copper in M. tuberculosis bacilli similar to that of 1-hydroxy-5-*R*-pyridine-2(1*H*)-thiones or disulfiram (41, 45); however, we cannot rule out an indirect effect of 11726172 on copper-dependent enzymes in M. tuberculosis.

Finally, we demonstrated that 11726172 is not a prodrug but is directly active against M. tuberculosis cells.

In conclusion, we have found a compound that is active *in vitro* and *in vivo* against nonreplicating cells and MDR clinical isolates of M. tuberculosis which could be used in combination with other antitubercular compounds because it does not exhibit antagonism. 11726172 has a peculiar copper-dependent mechanism of action that makes it very interesting for further preclinical evaluation.

## MATERIALS AND METHODS

### Bacterial strains and culture conditions.

M. tuberculosis H37Rv and H37Ra strains and M. bovis BCG were grown aerobically at 37°C in either Middlebrook 7H9 broth (Difco) supplemented with 0.2% glycerol, 0.05% Tween 80, or Tyloxapol at a final concentration of 0.05%, or in Sauton medium containing (per liter) 0.5 g KH_2_PO_4_, 1.4 g MgSO_4_ · 7H_2_O, 4 g l-asparagine, 60 mL glycerol, 0.05 g ferric ammonium citrate, 2 g sodium citrate, and 0.1 mL 1% ZnSO_4_, pH 7.0 (adjusted with 1 M NaOH), and supplemented with 0.05% Tween 80 or on Middlebrook 7H11 (Difco) plates supplemented with 0.5% glycerol, all supplemented with 10% OADC Middlebrook enrichment. The compounds were dissolved in DMSO (Sigma-Aldrich).

All the experiments with M. tuberculosis H37Rv were performed in a biosafety level-3 laboratory by authorized and trained researchers.

### Reagents and chemicals.

All reagents and solvents were purchased from commercial suppliers and used without further purification. ^1^H and ^13^C spectra were measured on a Bruker AC-300 (300 MHz, ^1^H) or a Bruker AC-200 (50 MHz, ^13^C) spectrometer. Chemical shifts were measured in DMSO-d_6_ or CDCl_3_, using tetramethylsilane as an internal standard, and reported as unit (parts per million) values. The following abbreviations are used to indicate multiplicity: s, singlet; d, doublet; t, triplet; m, multiplet; dd, doublet of doublets; brs, broad singlet; brm, broad multiplet. Mass spectra were recorded on a Finnigan MAT INCO 50 mass spectrometer (MS) (electron ionization, 70 eV) with direct injection. Melting points were determined on an Electrothermal 9001 apparatus (10°C per min) and are uncorrected.

### Synthesis of 11726172 and its derivatives.

4-Nitro-2,1,3-benzothiadiazol-5-yl 1,3-thiazolidine-3-carbodithioate (11726172) was synthesized as follows. A solution of 1.43 g (10 mmol) of 2-amino-4-chloroaniline in a mixture of 5.4 mL of thionyl chloride and 0.35 mL of concentrated sulfuric acid was refluxed for 1 h. The reaction mixture was cooled to 35°C, mixed with 4.3 mL of concentrated sulfuric acid, and stored for 20 min. This solution was treated with a mixture of 1.43 mL (32 mmol) of fumigating nitric acid and 2.2 mL of concentrated sulfuric acid at 20 to 25°C for 10 min. The reaction mixture was stored at 25 to 30°C for 30 min and poured into ice water. Gray precipitate was filtered off, washed with water and methanol, and recrystallized from methanol. The yield of 5-chloro-4-nitro-2,1,3-benzothiadiazole is 1.6 g (74%). The melting point (mp) was 145 to 147°C.

A suspension of 0.5 g (2.32 mmol) of 5-chloro-4-nitro-2,1,3-benzothiadiazole, 0.67 g (2.8 mmol) of 1,3-thiazolidine-3-carbodithioic acid sodium salt dihydrate in 25 mL of ethanol was stored for 45 min at room temperature. The reaction mixture was diluted with 100 mL of cold water, and the light-yellow precipitate was filtered off. The yield of 4-nitro-2,1,3-benzothiadiazol-5-yl 1,3-thiazolidine-3-carbodithioate 11726172 is 0.64 g (80%). The mp was 136 to 138°C (ethanol). MS (EI): *m/z* 344.4603 C_10_H_8_N_4_O_2_S_4_; ^1^H NMR (DMSO-d_6_): δ 8.23 (1H, d, *J *= 8.8 Hz, CH), 7.98 (1H, d, *J *= 8.8 Hz, CH), 4.27 (2H, m, CH_2_), 3.81 (2H, s, SCH_2_N), 2.52 (2H, m, CH_2_) ppm; ^13^C (DMSO-d_6_): δ 183.13, 166.09, 150.22, 146.55, 136.15, 127.79, 118.93, 76.01, 60.64, 29.18 ppm.

The synthesis of the other described derivatives was carried out according to the same procedure using the corresponding sodium salts of carbodithioic acid or amine in the presence of an equimolar amount of triethylamine.

4-Nitro-2,1,3-benzothiadiazol-5-yl thiomorpholine-4-carbodithioate (11726170) was synthesized. The mp was 149 to 151°C (ethanol). MS (EI): *m/z* 358.4869 C_11_H_10_N_4_O_2_S_4_; ^1^H NMR (DMSO-d_6_): δ 8.23 (1H, d, *J *= 8.8 Hz, CH), 7.99 (1H, d, *J *= 8.8 Hz, CH), 3.18 (4H, m, N(CH_2_)), 2.66 (4H, m, S(CH_2_)) ppm; ^13^C (DMSO-d_6_): δ 183.21, 166.09, 150.29, 146.55, 136.19, 125.78, 120.97, 56.52, 27.01 ppm.

4-Nitro-2,1,3-benzothiadiazol-5-yl 2-methyl-1,4-dioxa-8-azaspiro[4.5]decane-8-carbodithioate (11726171) was synthesized. The mp was 173 to 175°C (ethanol). MS (EI): *m/z* 412.5101 C_15_H_16_N_4_O_4_S_3_; ^1^H NMR (DMSO-d_6_): δ 8.23 (1H, d, *J *= 8.8 Hz, CH), 7.99 (1H, d, *J *= 8.8 Hz, CH), 4.36 (4H, m, N(CH_2_)), 4.31 (1H, m, CH), 4.18 (2H, m, CH_2_), 1.59 (4H, m, C(CH_2_)), 1.43 (3H, d, *J *= 4.3 Hz, CH_3_) ppm; ^13^C (DMSO-d_6_): δ 170.56, 166.12, 150.21, 147.01, 136.12, 127.64, 121.02, 106.21, 75.08, 70.51, 47.71, 42.40, 40.40, 18.51 ppm.

4-Nitro-2,1,3-benzothiadiazol-5-yl benzyl(isopropyl)dithiocarbamate (11726173) was synthesized. The mp was 132 to135°C (ethanol). MS (EI): *m/z* 404.5327 C_17_H_16_N_4_O_2_S_3_; ^1^H NMR (DMSO-d_6_): δ 8.28 (1H, d, *J *= 8.8 Hz, CH), 8.04 (1H, d, *J *= 8.8 Hz, CH), 7.52 (2H, m, 3CH), 7.11 (2H, m, 2CH), 4.51 (2H, s, CH_2_), 3.53 (1H, m, CH), 0.98 (6×H, d, *J *= 7.4 Hz, CH_3_) ppm; ^13^C (DMSO-d_6_): δ 185.12, 166.12, 150.23, 146.23, 136.80, 136.15, 129.00, 128.97, 126.66, 124.95, 54.67, 51.41, 19.97 ppm.

4-Nitro-2,1,3-benzothiadiazol-5-yl (4-chlorobenzyl)methyldithiocarbamate (11726174) was synthesized. The mp was 161 to 163°C (methanol). MS (EI): *m/z* 410.9243 C_15_H_11_ClN_4_O_2_S_3_; ^1^H NMR (DMSO-d_6_): δ 8.25 (1H, d, *J *= 8.8 Hz, CH), 8.01 (1H, d, *J *= 8.8 Hz, CH), 7.19 (2H, d, *J *= 7.6 Hz, 2CH), 7.05 (2H, d, *J *= 7.6 Hz, 2CH), 7.11 (2H, m, 2CH), 4.49 (2H, s, CH_2_), 3.59(3H, s, CH_3_) ppm; ^13^C (DMSO-d_6_): δ 184.65, 166.08, 150.21, 145.92, 136.45, 134.07, 131.72, 129.73, 129.41, 127.04, 122.69, 55.48, 41.06 ppm.

4-Nitro-2,1,3-benzothiadiazol-5-yl 3,4-dihydroisoquinoline-2(1*H*)-carbodithioate (11726175) was synthesized. The mp was 192 to195°C (ethanol). MS (EI): *m/z* 388.4902 C_16_H_12_N_4_O_2_S_3_; ^1^H NMR (DMSO-d_6_): δ 8.22 (1H, d, *J *= 8.8 Hz, CH), 8.00 (1H, d, *J *= 8.8 Hz, CH), 7.52 (1H, d, *J *= 8.2 Hz, CH), 7.24 (2H, m, CH=CH), 7.09 (2H, d, *J *= 8.2 Hz, 2CH), 3.87 (2H, s, CH_2_), 2.59 (2H, m, CH_2_) ppm; ^13^C (DMSO-d_6_): δ 186.56, 166.13, 150.27, 145.76, 140.88, 140.01, 136.37, 129.51, 127.93, 127.56, 126.54, 123.83, 52.88, 51.82, 29.81 ppm.

4-Nitro-2,1,3-benzothiadiazol-5-yl 4-(4-fluorophenyl)piperazine-1-carbodithioate (11726176) was synthesized. The mp was 107to 109°C (ethyl acetate/hexane). MS (EI): *m/z* 435.5221 C_17_H_14_FN_5_O_2_S_3_; ^1^H NMR (DMSO-d_6_): δ 8.24 (1H, d, *J *= 8.8 Hz, CH), 7.98 (1H, d, *J *= 8.8 Hz, CH), 6.68 (4H, m, 4CH), 3.22 (4H, m, 2CH_2_), 2.96 (4H, m, 2CH_2_) ppm; ^13^C (DMSO-d_6_): δ 187.17, 166.11, 160.01, 153.01, 150.16, 146.18, 144.55, 136.78, 131.12, 120.97, 115.45, 114.76, 114.63, 114.47, 51.17, 50.73 ppm.

4-Nitro-5-thiomorpholin-4-yl-2,1,3-benzothiadiazole (11726177) was synthesized. The mp was 97 to 101°C (ethanol). MS (EI): *m/z* 282.3442 C_10_H_10_N_4_O_2_S_2_; ^1^H NMR (DMSO-d_6_): δ 7.93 (1H, d, *J *= 8.8 Hz, CH), 7.02 (1H, d, *J *= 8.8 Hz, CH), 3.92 (4H, m, N(CH_2_)_2_), 2.47 (4H, m, S(CH_2_)_2_) ppm; ^13^C (DMSO-d_6_): δ 161.06, 145.77, 130.43, 126.31, 124.98, 108.09, 48.93, 48.87, 26.12, 25.97 ppm.

4-Nitro-*N*-(2-thienylmethyl)-2,1,3-benzothiadiazol-5-amine (11726178) was synthesized. The mp was 139 to 141°C (ethanol). MS (EI): *m/z* 292.3391 C_11_H_8_N_4_O_2_S_2_; ^1^H NMR (DMSO-d_6_): δ 10.03 (1H, br s, NH), 7.84 (1H, d, *J *= 8.8 Hz, CH), 7.31 (2H, m, CH=CH), 7.03 (1H, d, *J *= 6.4 Hz, CH), 7.02 (1H, d, *J *= 8.8 Hz, CH), 4.58 (2H, s, CH_2_) ppm; ^13^C (DMSO-d_6_): δ 160.59, 152.23, 142.72, 134.63, 127.28, 126.32, 125.93, 121.64, 120.99, 108.07, 46.57 ppm.

*N*-{2-[4-(Methylthio)phenyl]ethyl}-4-nitro-2,1,3-benzothiadiazol-5-amine (11726179) was synthesized. The mp was 154 to 156°C (methanol). MS (EI): *m/z* 346.4294 C_15_H_14_N_4_O_2_S_2_; ^1^H NMR (DMSO-d_6_): δ 9.86 (1H, br s, NH), 7.80 (1H, d, *J *= 8.8 Hz, CH), 7.14 (2H, d, *J *= 7.3 Hz, 2CH), 7.02 (1H, d, *J *= 8.8 Hz, CH), 6.88 (2H, d, *J *= 7.3 Hz, 2CH), 4.04 (2H, m, CH_2_), 3.11 (2H, m, CH_2_), 2.41 (3H, s, SCH_3_) ppm; ^13^C (DMSO-d_6_): δ 160.47, 152.29, 140.78, 134.29, 133.72, 130.04, 129.98, 126.43, 126.13, 122.51, 119.84, 108.98, 43.58, 37.01, 16.06 ppm.

5-(4-Benzylpiperazin-1-yl)-4-nitro-2,1,3-benzothiadiazole (11726180) was synthesized. The mp was 87 to 89°C (methanol). MS (EI): *m/z* 355.4154 C_17_H_17_N_5_O_2_S; ^1^H NMR (DMSO-d_6_): δ 7.63 (1H, d, *J *= 8.8 Hz, CH), 7.26 (3H, m, 3CH), 7.19 (3H, m, 3CH), 7.05 (1H, d, *J *= 8.8 Hz, CH), 3.67 (4H, m, N(CH_2_)_2_), 3.61 (2H, s, CH_2_), 2.52 (4H, m, 2CH_2_) ppm; ^13^C (DMSO-d_6_): δ 161.15, 145.77, 139.40, 134.52, 132.21, 129.21, 128.51, 126.96, 99.91, 119.84, 108.98, 62.96, 46.47, 45.98 ppm.

4-{2-[(4-Nitro-2,1,3-benzothiadiazol-5-yl)amino]ethyl}benzenesulfonamide (11726181) was synthesized. The mp was 173 to 176°C (ethanol). MS (EI): *m/z* 379.4163 C_14_H_13_N_5_O_2_S_2_; ^1^H NMR (DMSO-d_6_): δ 7.78 (1H, d, *J *= 8.8 Hz, CH), 7.69 (2H, d, *J *= 6.2 Hz, 2CH), 7.16 (2H, d, *J *= 6.2 Hz, 2CH), 6.89 (1H, d, *J *= 8.8 Hz, CH), 4.06 (2H, m, NCH_2_), 3.08 (2H, m, CH_2_) ppm; ^13^C (DMSO-d_6_): δ 161.03, 152.23, 145.82, 141.96, 133.74, 130.29, 127.25, 122.48, 119.83, 108.95, 43.57, 37.06 ppm.

### Macromolecular synthesis assays with M. tuberculosis H37Ra treated with 11726172.

The macromolecular synthesis assays with M. tuberculosis H37Ra treated with 11726172 were performed as described previously (19). Briefly, when the optical density at 600 nm (OD_600_) of the culture reached 0.2, 100-μL aliquots were transferred to 2-mL Eppendorf tubes containing the tested compound at increasing concentration (1, 1.5, 1.75, 2, 2.5 μg/mL dissolved in 2 mL of DMSO) and 5 μL of ^14^C-labeled substrates ([^14^C]acetate [specific activity, 110 mCi/mmol, American Radiolabeled Chemicals, Inc.], [^14^C]leucine [specific activity, 328 mCi/mmol; Moravek Inc.], [^14^C]methionine [specific activity, 55 mCi/mmol; American Radiolabeled Chemicals, Inc.], or [^14^C]uracil [specific activity, 53 mCi/mmol; American Radiolabeled Chemicals, Inc.] in a final concentration of 0.5 μCi/mL) a sample not treated with 11726172 was also included. After 24 h of static incubation at 37°C, the cultures were precipitated with 5% trichloroacetic acid (TCA) for 1 h on ice, harvested by centrifugation (14,000 × *g*, 10 min, 4°C), and washed twice with 5% TCA, and incorporation of ^14^C-labeled substrates was quantified by scintillation spectrometry.

### Lipid analysis of M. tuberculosis H37Ra and H37Rv treated with 11726172.

Metabolic labeling of the cells of M. tuberculosis H37Ra or M. tuberculosis H37Rv with [^14^C]acetate was performed as described above. The final concentrations of tested compound 11726172 in medium for M. tuberculosis H37Rv treatment were 0, 0.25, 0.5, 1, and 2.5 μg/mL. A not treated sample was also included.

The lipids were isolated from [^14^C]acetate-labeled cells and analyzed by thin-layer chromatography (TLC) as follows: the lipids were extracted by the addition of 3 mL of chloroform-methanol (2/1, vol/vol) to 100 μL of culture, followed by 2 h of incubation at 65°C. A 400-μL volume of water was added to each sample to make a biphasic solution, the samples were centrifuged, and the lower organic phase was removed and dried under nitrogen. The lipid samples were washed as described by Folch et al. (46), and the final dried extract was resolved in 50 μL of chloroform-methanol (2/1, vol/vol). Five microliters was loaded on each line of the TLC plate (silica gel plates F_254_) and developed in solvent I (chloroform-methanol-water [20/4/0.5]) or solvent II (chloroform-methanol-ammonium hydroxide-water [65/25/0.5/4]). The lipids were visualized using an Amersham Typhoon biomolecular imager.

Fatty acid methyl esters (FAME) and mycolic acid methyl esters (MAME) were prepared from whole cells from 100-μL cultures as previously described (47). Dried extracts were dissolved in 50 μL of chloroform-methanol (2/1, vol/vol), and 5 μL was loaded on the TLC plates. FAME and different forms of MAME were separated by three runs in solvent III *n*-hexane–ethyl acetate (95/5) and detected by autoradiography.

### Metabolic transformation of 11726172 in M. bovis BCG cultures.

To identify the products of 11726172 transformation in a mycobacterial environment, 200 mL of M. bovis BCG culture was grown statically at 37°C in 7H9 medium supplemented with 0.05% Tween 80 and 10% OADC Middlebrook enrichment. When an OD_600_ of 0.7 was reached, 10 mg of compound (corresponding to 100× MIC) was added, and the culture was incubated for a further 16 h and then extracted with chloroform (100 mL, three times). The organic phase obtained was evaporated, and the residues were resuspended in hexane-ethyl acetate (6:4, vol/vol) and subjected to flash column chromatography (Merck SiO_2_ 60, 230 to 400 mesh). Visualization of metabolites was achieved under UV light at a wavelength of 254 nm.

The isolated metabolites were analyzed by heated electrospray ionization-mass spectrometry (HESI-MS), performed with an MS from Thermo Scientific (Milan, Italy) LTQ XL HESI-MS/MS system using direct injection of purified fractions.

Details of the system were as follows. For the HESI probe, gas = N_2_, *T* = 90°C, voltage = 3.3 kV; capillary *T* = 275°C, voltage = 48 V, and tube lens = 70 V. For the tune settings, multipole 00 offset = 2.5 V, lens 0 = −4.28 V, multipole 0 offset = −5.12 V, lens 1 = −8.91, gate lens = −66.1 V, multipole 1 offset = −6.4 V, multipole RF amplitude (p-p) = 400 V, and front lens = −6.0 V. Settings for MS-MS and MS^3^ were as follows: detection by CID (collision-induced dissociation); isolation width, ±2 dalton; activation Quadrupole, 0.250; activation time, 30.0 ms; isolation width for quantitation, ±2.5 d.

### Determination of MIC.

The drug susceptibility of M. tuberculosis H37Rv strains was determined using the resazurin microtiter assay (REMA), as previously described (13). To study metal homeostasis, sterile solutions of CuSO_4_, NiSO_4_, CoCl_2_, and ZnSO_4_ in the concentration range of 5 to 50 μM were added to Sauton medium supplemented with 10% OADC Middlebrook enrichment to determine the influence of metal cations on MIC determination. A growth control containing no compound and a sterile control without inoculum were also included. After 7 days of incubation at 37°C, 10 μL of resazurin (0.05%, wt/vol) was added and fluorescence was measured after an additional 24 h of incubation with a Fluoroskan microplate fluorometer (Thermo Fisher Scientific; excitation = 544 nm, emission = 590 nm). Bacterial viability was calculated as a percentage of resazurin turnover in the absence of compound. The assay was repeated at least three times.

### Determination of bactericidal activity against dormant NC M. tuberculosis.

Bactericidal activity against dormant nonculturable (NC) M. tuberculosis H37Rv was examined using the most probable number (MPN) assay. Briefly, 2 × 10^7^ dormant NC M. tuberculosis H37Rv bacteria obtained under potassium deficiency (48) were treated with various concentrations of the compound for 7 days (200 rpm, 37°C). To estimate the proportion of viable bacteria, cells were washed after treatment with 11726172 by a fresh albumin-dextrose-catalase (ADC)-supplemented Sauton medium, and then 10-fold bacterial dilutions were prepared resuspended in ADC-supplemented Sauton medium diluted 1:1 (vol/vol; final glycerol concentration, 0.6%) and seeded into 48-well Corning microplates, which were incubated statically at 37°C for 30 days (15). Wells with visible bacterial growth were considered positive. The number of bacteria (MPN values) was calculated using standard statistical methods (49). Then, MPN values after a 7-day exposure to the compounds were compared with MPN values of untreated cells in order to measure the bactericidal effect of each concentration of the compound against dormant bacilli.

### RNA extraction.

Triplicates of M. tuberculosis H37Rv cultures grown to mid-exponential phase were treated with two 11726172 concentrations, corresponding to 10× and 30× MIC (2.5 μg/mL and 7.5 μg/mL, respectively). Triplicates of not-treated cultures were included as a control. After 4 h of treatment, cells were pelleted, flash frozen in liquid nitrogen, and stored at −80°C until use. RNA was extracted as previously described (50).

### RNA-seq.

RNA-seq was performed by IGA Technology Service, Udine, Italy (https://igatechnology.com/), using an Illumina platform. Ribosomal transcript depletion was performed using the Universal Prokaryotic RNA-Seq, Prokaryotic AnyDeplete library preparation kit (NuGEN, San Carlos, CA) according to the protocol provided by the supplier. RNA samples were quantified and quality tested using the Agilent 2100 bioanalyzer RNA assay (Agilent Technologies, Santa Clara, CA). Strand-specific RNA-seq libraries (library type, fr-secondstrand) were then generated and checked with both a Qubit 2.0 fluorometer (Invitrogen, Carlsbad, CA) and an Agilent bioanalyzer DNA assay. Libraries were then sequenced on single-end 75-bp mode on a NextSeq 500 system (Illumina, San Diego, CA). Libraries were then sequenced on a NextSeq 500 machine (Illumina, Waltham, CA), generating a total of 439 million single-end reads of 75 nucleotides in length.

Reads were quality checked using the FASTQC tool (https://qubeshub.org/resources/fastqc) and preprocessed by Trimmomatic v0.38 (51) to trim the adaptor and remove low-quality sequences. The resulting clean reads were mapped to the M. tuberculosis H37Rv reference genome (RefSeq accession no. NC_000962.3) using Bowtie2 v2.2.6 (52). Gene expression estimates were made as raw read counts using featureCounts v1.6.4 (53) and summarized at the coding DNA sequence (CDS) level.

Normalization of raw read counts and differential expression analysis were performed using DeSeq2 v1.24.0 (54). Differentially expressed genes (DEGs) were defined using the following criteria: log2 fold change of ≥| 2.5 | (sample group/control) and an FDR of <0.05.

Last, to account for biological and technical variability within different conditions and to increase statistical power and accuracy in detecting differentially expressed genes, meta-analyses of RNA-seq data were performed using the R package MetaRNASeq v1.0.2 (55). DEGs were defined using the following criteria: up- or downregulated in the same direction, inverse normal and Fisher method *P* values of <0.05. Differences in the enrichment of gene ontology (GO) categories and KEGG pathways for the DEGs were analyzed using the DAVID (v6.8) functional annotation analysis tool (https://david.ncifcrf.gov/) (56), with default settings. The distribution of M. tuberculosis genes to functional categories was also performed based on the TubercuList (30).

### Quantitative real-time PCR.

Purified total RNA was retrotranscribed using a QuantiTect reverse transcription kit (Qiagen) in accordance with the manufacturer’s recommendations. Real-Time PCR was performed as previously described (23). Primers are listed in Table S2 in the supplemental material.

10.1128/msphere.00369-22.2 TABLE S2 Primers used in real-time PCR experiments. Download Table S2, DOCX file, 0.01 MB. Copyright © 2022 Salina et al. 2022 Salina et al. https://creativecommons.org/licenses/by/4.0/ This content is distributed under the terms of the Creative Commons Attribution 4.0 International license.

### Determination of metal content.

For preparation of M. tuberculosis H37Rv lysate, cultures with a starting OD_600_ of 0.005 were cultivated in Sauton medium supplemented with OADC (10%) and Tween 80 (0.05%). When the OD_600_ reached 0.5, 50 μM concentration each of CuSO_4_, NiSO_4_, CoCl_2_, and ZnSO_4_, with or without compound 11726172 at a final concentration of 7.5 μg/mL (30× MIC), were added to the cultures, which were incubated for the next 2 h. DMSO (0.1%) served as a control. Cells were harvested by centrifugation (4,000 × *g*, 15 min, 4°C), washed twice with 10 mM phosphate-buffered saline (PBS), resuspended in 10 μM PBS (25 mg/mL of wet weight), disintegrated in a BeadBeater (Biospec, USA) with 100-mm zirconia beads, and centrifuged (4,000 × *g*, 45 min, 4°C) to clear cell lysates from cell debris. The inductively coupled plasma (ICP)-MS measurements for M. tuberculosis lysates were carried out with an Aurora M90 quadrupole ICP-MS instrument (Bruker Corp., USA) equipped with a MicroMist low-flow nebulizer. A series of Cu^2+^, Ni^2+^, Co^2+^, and Zn^2+^ standard solutions (0.1 to 5.0 ppb in 1% [vol/vol] HNO_3_) was prepared before each experiment. All samples were prepared in triplicate. Quantum software (Bruker Corp., v 3.1 b1433) was used for data collection and processing. The quantification of metal concentrations was carried out by using a calibration curve.

### CBAs.

To determine pairwise drug interactions, we used checkerboard assays (CBAs). Briefly, serial dilutions of drug partners were performed at different combination ratios in a 96-well black plate (FluoroNunc; Thermo Fisher, Waltham, MA, USA), and then 100 μL of a bacterial inoculum, approximately of 10^5^ CFU/mL, was added. Growth controls containing no compound and sterile controls without inoculum were also included. After 7 days of incubation at 37°C, 10 μL of resazurin (0.025%, wt/vol) was added to each well. The effect of the combination was measured using growth inhibition as the endpoint readout after 1 day of further incubation, by means of a Fluoroskan microplate fluorometer (Thermo Fisher Scientific, Waltham, MA, USA; excitation = 544 nm, emission = 590 nm). Then, the FICI value was calculated. The MIC of drug A in the presence of drug B divided by the MIC of drug A alone [FIC_A_ = (MIC_A(B)/_MIC_A_)] is defined as the FIC of drug A (FIC_A_) and vice versa [FIC_B_ = (MI_CB(A)_/MIC_B_)]. The sum of these values gives the final parameter, FICI. “Synergy” is defined as a ≥4-fold reduction in the MICs of both compounds in combination compared to their MICs alone (FICI ≤ 0.5); “no interaction” is defined as when the MIC of one of the compounds remained in the range of 12× MIC to 4× MIC (FICI > 0.5 to 4); and “antagonism” is defined as when the MIC of both compounds is at least 4-fold higher than the activity of the compounds alone (FICI > 4.0) (57).

### Murine infection model, treatment, and assessment of efficacy.

All animal experiments were conducted under protocols approved by the Institutional Animal Care and Use Committee at the Central Research Institute of Tuberculosis, Moscow (IACUC protocol 076-2020), according to the institution’s guidelines for animal use, the state industry standards GOST 33215-2014 and GOST 33216-2014 (in harmony with the European Directive 2010/63/ЕС). The State Veterinary Service of Russian Federation approved experiments (authorization no. 754 0219882).

Male BALB/c/Cit mice, aged 5 to 6 weeks, were obtained from the Central Research Institute of Tuberculosis, Moscow, Russia. Animals were infected by intravenous injection of M. tuberculosis Н37Rv at a dose of 5 × 10^6^ CFU/mouse into the lateral tail vein. Starting from day 7 after infection and for a further 4 weeks, mice were treated with either 100 mg/kg of 11726172 or 25 mg/kg of INH (control drug), administered by gavage 5 days a week. A not treated group of mice was included as negative control.

Mice were sacrificed by cervical dislocation 2 days after the last treatment (for elimination of residue drugs from blood). To determine the efficacy of each treatment, macroscopic changes in animal parenchymatous organs were taken into consideration. Then, the lungs were homogenized in 2 mL of saline buffer, a series of 10-fold dilutions in saline buffer was prepared, and 50 μL of each dilution was plated onto Dubos agar plates. The plates were incubated for 21 days at 37°С, and then the numbers of CFU were determined by counting.

### Data availability.

The RNA-seq data have been deposited in the NCBI Sequence Read Archive database under BioProject accession number PRJNA802089.
