# Supplementary material for: A New Benzothiazolthiazolidine Derivative, 11726172, Is Active In Vitro, In Vivo, and against Nonreplicating Cells of Mycobacterium tuberculosis
Source: mSphere. 2022 Nov 15;7(6):e00369-22. doi: 10.1128/msphere.00369-22 (PMC9769805; doi:10.1128/msphere.00369-22)
Supplement: TABLE S1 [file msphere.00369-22-s0001.docx]

| **Gene** | **log2FoldChange** | ***P*-value** | **padj** | **Functional category** |
| --- | --- | --- | --- | --- |
| *ArsC* | 4,518071112 | 2,14E-22 | 3,77E-20 | Cell wall and cell processes |
| *CtpG* | 4,405768741 | 1,14E-18 | 1,19E-16 | Cell wall and cell processes |
| *Rv1972* | 3,94044767 | 7,76E-19 | 8,60E-17 | Cell wall and cell processes |
| *Rv1671* | 3,914358516 | 1,68E-10 | 5,34E-09 | Cell wall and cell processes |
| *LpqS* | 3,774893373 | 1,33E-12 | 6,44E-11 | Cell wall and cell processes |
| *Rv2698* | 3,646690147 | 7,36E-18 | 7,14E-16 | Cell wall and cell processes |
| *Rv0180c* | 3,462145238 | 2,04E-13 | 1,18E-11 | Cell wall and cell processes |
| *Rv2963* | 3,440360891 | 4,21E-16 | 3,14E-14 | Cell wall and cell processes |
| *CtpV* | 3,43796012 | 1,38E-16 | 1,11E-14 | Cell wall and cell processes |
| *Rv2025c* | 3,298868065 | 1,46E-25 | 4,34E-23 | Cell wall and cell processes |
| *Rv3054c* | 8,028457729 | 1,21E-42 | 1,17E-39 | Conserved hypotheticals |
| *Rv2641* | 7,776595731 | 2,22E-48 | 2,86E-45 | Conserved hypotheticals |
| *Rv1673c* | 7,170091047 | 4,42E-29 | 1,43E-26 | Conserved hypotheticals |
| *Rv3178* | 6,62246002 | 2,62E-67 | 5,08E-64 | Conserved hypotheticals |
| *Rv0326* | 6,484090414 | 4,44E-20 | 5,56E-18 | Conserved hypotheticals |
| *Rv3463* | 5,717553237 | 1,55E-21 | 2,50E-19 | Conserved hypotheticals |
| *Rv0140* | 5,643142982 | 1,58E-19 | 1,86E-17 | Conserved hypotheticals |
| *Rv1767* | 4,495728779 | 2,01E-20 | 2,68E-18 | Conserved hypotheticals |
| *Rv0141c* | 4,403819157 | 1,47E-15 | 9,83E-14 | Conserved hypotheticals |
| *Rv1993c* | 4,357376583 | 7,64E-36 | 4,23E-33 | Conserved hypotheticals |
| *Rv2466c* | 4,338320456 | 7,41E-12 | 3,06E-10 | Conserved hypotheticals |
| *Rv3353c* | 4,069287459 | 2,40E-21 | 3,58E-19 | Conserved hypotheticals |
| *Rv0325* | 3,872814579 | 1,45E-10 | 4,77E-09 | Conserved hypotheticals |
| *Rv2822c* | 3,86098771 | 1,32E-11 | 5,06E-10 | Conserved hypotheticals |
| *Rv0740* | 3,704388866 | 3,95E-12 | 1,72E-10 | Conserved hypotheticals |
| *Rv0142* | 3,621254961 | 9,37E-17 | 7,87E-15 | Conserved hypotheticals |
| *Rv0990c* | 3,326057377 | 1,71E-10 | 5,40E-09 | Conserved hypotheticals |
| *Rv0991c* | 3,310931575 | 4,71E-07 | 6,95E-06 | Conserved hypotheticals |
| *Rv3222c* | 3,101722956 | 1,04E-11 | 4,20E-10 | Conserved hypotheticals |
| *Rv2699c* | 3,010397266 | 1,29E-11 | 5,02E-10 | Conserved hypotheticals |
| *Rv3122* | 3,008448456 | 9,33E-10 | 2,62E-08 | Conserved hypotheticals |
| *Rv1875* | 3,005266222 | 9,86E-10 | 2,75E-08 | Conserved hypotheticals |
| *Rv2706c* | 2,88680234 | 6,72E-08 | 1,24E-06 | Conserved hypotheticals |
| *Rv0329c* | 2,820597346 | 4,23E-09 | 1,00E-07 | Conserved hypotheticals |
| *Rv0968* | 2,803809861 | 2,49E-12 | 1,14E-10 | Conserved hypotheticals |
| *Rv0347* | 2,799508287 | 2,42E-08 | 4,92E-07 | Conserved hypotheticals |
| *Rv1954c* | 2,796090532 | 3,59E-05 | 0,000313428 | Conserved hypotheticals |
| *Rv3269* | 2,76971718 | 3,05E-09 | 7,46E-08 | Conserved hypotheticals |
| *Rv2823c* | 2,584897258 | 1,80E-08 | 3,83E-07 | Conserved hypotheticals |
| *Rv1461* | 2,532006046 | 4,59E-07 | 6,79E-06 | Conserved hypotheticals |
| *Rv2824c* | 2,526551809 | 1,24E-07 | 2,10E-06 | Conserved hypotheticals |
| *RshA* | 3,442741193 | 7,41E-12 | 3,06E-10 | Information pathways |
| *Rv0560c* | 7,213225532 | 1,25E-79 | 4,85E-76 | Intermediary metabolism and respiration |
| *Rv3174* | 6,899626007 | 1,63E-36 | 1,05E-33 | Intermediary metabolism and respiration |
| *Cyp135A1* | 6,561143496 | 1,61E-21 | 2,50E-19 | Intermediary metabolism and respiration |
| *Rv1050* | 5,988700049 | 2,99E-23 | 5,79E-21 | Intermediary metabolism and respiration |
| *TrxC* | 5,483632925 | 2,58E-21 | 3,70E-19 | Intermediary metabolism and respiration |
| *TrxB2* | 5,093034989 | 1,73E-20 | 2,39E-18 | Intermediary metabolism and respiration |
| *Rv3175* | 5,067351391 | 8,94E-25 | 2,31E-22 | Intermediary metabolism and respiration |
| *Rv0331* | 4,556868683 | 5,59E-14 | 3,49E-12 | Intermediary metabolism and respiration |
| *Rv2962c* | 4,327218426 | 6,03E-23 | 1,11E-20 | Intermediary metabolism and respiration |
| *TrxB1* | 4,026790156 | 1,06E-12 | 5,33E-11 | Intermediary metabolism and respiration |
| *CysO* | 3,95192163 | 1,18E-13 | 7,07E-12 | Intermediary metabolism and respiration |
| *Rv1334* | 3,915688946 | 1,39E-12 | 6,67E-11 | Intermediary metabolism and respiration |
| *Rv0793* | 3,837849182 | 9,35E-16 | 6,36E-14 | Intermediary metabolism and respiration |
| *MoeB1* | 3,765327428 | 2,15E-14 | 1,36E-12 | Intermediary metabolism and respiration |
| *ThiX* | 3,710388471 | 1,75E-16 | 1,39E-14 | Intermediary metabolism and respiration |
| *Rv0794c* | 3,416868418 | 2,41E-13 | 1,37E-11 | Intermediary metabolism and respiration |
| *cysK2* | 3,273069865 | 5,59E-13 | 2,97E-11 | Intermediary metabolism and respiration |
| *Rv0953c* | 3,208001113 | 3,10E-11 | 1,14E-09 | Intermediary metabolism and respiration |
| *Rv0846c* | 3,130370101 | 1,28E-11 | 5,02E-10 | Intermediary metabolism and respiration |
| *ClpC2* | 3,007233048 | 8,37E-16 | 5,98E-14 | Intermediary metabolism and respiration |
| *PrpC* | 2,959905943 | 1,40E-09 | 3,68E-08 | Intermediary metabolism and respiration |
| *CysM* | 2,905616256 | 1,21E-09 | 3,27E-08 | Intermediary metabolism and respiration |
| *Rv2454c* | 2,864321332 | 2,64E-08 | 5,27E-07 | Intermediary metabolism and respiration |
| *PrpD* | 2,611847375 | 3,77E-07 | 5,71E-06 | Intermediary metabolism and respiration |
| *Rv1188* | 2,562357693 | 3,38E-08 | 6,55E-07 | Intermediary metabolism and respiration |
| *MoaE1* | 2,556977018 | 1,44E-07 | 2,39E-06 | Intermediary metabolism and respiration |
| *PapA4* | 3,565702408 | 4,51E-12 | 1,94E-10 | Lipid metabolism |
| *PPE29* | 3,502501932 | 1,69E-08 | 3,65E-07 | PE/PPE |
| *PPE15* | 2,796729217 | 1,22E-09 | 3,27E-08 | PE/PPE |
| *Rv2642* | 6,420973709 | 2,07E-39 | 1,60E-36 | Regulatory proteins |
| *Rv1674c* | 6,373063975 | 9,51E-25 | 2,31E-22 | Regulatory proteins |
| *Rv1049* | 6,017140395 | 1,12E-23 | 2,42E-21 | Regulatory proteins |
| *CmtR* | 5,676652872 | 6,69E-34 | 3,25E-31 | Regulatory proteins |
| *Rv2640c* | 4,518659411 | 1,55E-18 | 1,58E-16 | Regulatory proteins |
| *CmtR* | 4,353529367 | 8,24E-25 | 2,28E-22 | Regulatory proteins |
| *CsoR* | 3,535498364 | 2,03E-13 | 1,18E-11 | Regulatory proteins |
| *Rv0328* | 3,241144441 | 3,60E-10 | 1,09E-08 | Regulatory proteins |
| *Rv0196* | 2,629325655 | 6,16E-07 | 8,82E-06 | Regulatory proteins |
| *Rv3177* | 6,100366192 | 4,52E-30 | 1,95E-27 | Virulence, detoxification, adaptation |
| *MesT* | 4,091595889 | 1,19E-17 | 1,10E-15 | Virulence, detoxification, adaptation |
| *ClpB* | 4,039971344 | 1,18E-09 | 3,23E-08 | Virulence, detoxification, adaptation |
| *MymT* | 3,871133181 | 6,58E-16 | 4,82E-14 | Virulence, detoxification, adaptation |
| *YrbE3A* | 3,86375075 | 2,40E-16 | 1,82E-14 | Virulence, detoxification, adaptation |
| *KatG* | 3,332098999 | 1,46E-12 | 6,90E-11 | Virulence, detoxification, adaptation |
| *YrbE3A* | 2,88996788 | 2,12E-05 | 0,000198383 | Virulence, detoxification, adaptation |
| *HigB* | 2,687673125 | 3,97E-06 | 4,46E-05 | Virulence, detoxification, adaptation |
| *MazE8* | -4,193542346 | 9,25E-17 | 7,87E-15 | Virulence, detoxification, adaptation |
| *Rv0047c* | -2,843151703 | 1,66E-08 | 3,59E-07 | Conserved hypotheticals |
| *Rv0430* | -2,707134436 | 2,84E-08 | 5,62E-07 | Conserved hypotheticals |
| *Rv1632c* | -2,57419872 | 4,23E-10 | 1,26E-08 | Conserved hypotheticals |
