# Supplementary material for: A New Benzothiazolthiazolidine Derivative, 11726172, Is Active In Vitro, In Vivo, and against Nonreplicating Cells of Mycobacterium tuberculosis
Source: mSphere. 2022 Nov 15;7(6):e00369-22. doi: 10.1128/msphere.00369-22 (PMC9769805; doi:10.1128/msphere.00369-22)
Supplement: TABLE S2 [file msphere.00369-22-s0002.docx]

| Oligonucletide name | Sequence 5'→3' | Purpose |
| --- | --- | --- |
| RTsigAF | gatgacgacgaggagat | qPCR of sigA |
| RTsigAR | gccgatctgtttgaggta |  |
| GD27RTcyp135f | cccaaaagtcaaggccga | qPCR of cyp135A1 |
| GD28RTcyp135r | gtctatcttggtttggtt |  |
| GD29RTtrxCf | tgtgctggttgacttttg | qPCR of trxC |
| GD30RTtrxCr | aacaagatcagggtaggg |  |
| GD31RTmazE8f | tgttatgccgtccgcatt | qPCR of mazE8 |
| GD31RTmazE8r | ggtccaaggaaaacaatg |  |
